# The Hospital

**Published:** 1902-04-12

**Authors:** 


### The Hospital.

### Nursing Section.

Contributions for this Section of "The Hospital" should be addressed to the Editor, "The Hospital" Nursing Section, 28 & 29 Southampton Street, Strand, London, W.C.

No. 811.—Vol. XXXII. *SATURDAY, APRIL* 12, 1902.

### Notes on News from the Nursing World.

### ROYAL NATIONAL PENSION FUND FOR NURSES.

The annual general meeting of the members of the Royal National Pension Fund for Nurses will be held at River Plate House, Finsbury Circus, London, E.C. (close to the offices of the Fund), on Thursday, 24th April, 1902, at 4 p.m. Any policy holder of the Fund who wishes to be present will be welcomed by the Council.

### THE MATRON-IN-CHIEF OF THE MILITARY NURSING SERVICE.

The matron-in-chief of Queen Alexandra's Imperial Military Nursing Service has arrived in England to take up her duties, which are of a very important and responsible character. For example, in her hands rests the obligation of making appointments, deciding upon promotions and retirements, and organising an adequate staff of special nurses who will be detailed for duty in cases of emergency, or for service in the smaller hospitals. It has been a matter of some comment that a lady whose name is widely known and esteemed in connection with Army nursing was not nominated to the post which has been bestowed upon Miss Browne temporarily; but we understand that her claims were far from being overlooked; perhaps, indeed, it may turn out that consideration for her had something to do with the temporary nature of the present appointment.

### A WOMAN'S ATTACK ON NURSES.

Nothing is easier than to draw up an indictment of a general character against a class of the community, and nothing is more difficult to refute, because the authors of such indictments are usually as sparing of their facts and figures as they are lavish in their invective. It is true that this renders their conclusions practically valueless, but it saves them from the complete exposure of ignorance and prejudice which they would otherwise have to endure. Miss M. F. Johnston, who has been allowed to air her opinions in an article entitled "The Case against Hospital Nurses" in the current number of *The Nineteenth Century* indulges in a tirade against hospital nurses. But the citation of two sentences should be sufficient to show that she has no sort of claim to a hearing. Speaking, not of a few, not even of a minority, of hospital nurses, she says, "They are only too generally wholly inconsiderate in the demands they make and offensive in their general behaviour. . . . Their callousness to suffering and the indifference they display even in the hour of death amount almost to brutality." This is an example of the serious nature of her charges. The worst offences, as well as the more trivial faults are, however, Miss Johnston asserts, "characteristic of the profession as a whole, and the exceptions are in a small minority." After this, her attempt to prove that negligent sisters and indifferent matrons are largely responsible for the shortcomings and offences of nurses, does not merit any notice. Of course, we all know that neither matrons nor sisters are perfect, and that hospital and other nurses are not free from the shortcomings of their sex. But an effort, however futile and grotesque, to hold up a noble profession to contumely is a curious mission for a woman to impose upon herself, and we print with pleasure in another page a rejoinder from a well-known matron of varied experience and unquestionable knowledge.

### THE WAR NURSES.

The *Canada* arrived on April 7th from South Africa, bringing the following nursing sisters:—T. Overbeck, A.N.S.R., acting-superintendent, who requires three months' leave and returns to South Africa; M. Hamer and L. E. Walker, time-expired; N. S. L. Bond, requires three months' leave and returns to South Africa. The three last mentioned are described as "civil nurses." On the 3rd of this month came four nursing sisters on board the *Dilwara*, viz. S. J. Browne, A.N.S., matron-in-chief of Queen Alexandra's Military Nursing Service; A. M. Rose, A.N.S.R., requiring one month; J. C. S. Spence, A.N.S.R., requiring two months'; and M. J. Harrower, A.N.S.R., requiring one month's leave.

### PAUPER "NURSES" IN CUMBERLAND AND WESTMORLAND.

A fortnight ago we published some figures from a remarkable return recording the number of sick inmates in the workhouses of Lancashire, and showing the number of nurses and pauper attendants employed in their care. The return, though that of Mr. Jenner Fust, general inspector of the Lancashire District under the Local Government Board, was not issued from headquarters. This, however, in no way detracts from its importance, and we are now able to quote the figures which Mr. Jenner Fust has compiled relating to the workhouses in part of Cumberland and the whole of Westmorland. Taking Cumberland first, we learn that in each of the five unions enumerated, namely, Bootle, Cockermouth, Penrith, Whitehaven, and Wigton, pauper attendants are employed in nursing. In Bootle there are two to one paid nurse, in Cockermouth ten to one paid nurse, in Penrith two to one paid nurse, in Whitehaven ten to two paid nurses, and in Wigton four to two paid nurses. For Westmorland the figures are:—East ward, one paid nurse and no pauper attendant; West ward, no paid nurse, and two pauper assistants; Kendal, one paid nurse and one pauper assistant; Milnthorpe, one paid nurse and two pauper assistants. In other words, the entire return gives a total of ten paid nurses, of whom only four, we see, were trained for three years, as against 33 pauper attendants. But after the figures for Lancashire, we are not surprised.

### PAUPER "NURSES" IN THE MIDLANDS.

Mr. E. B. Wethered, General Inspector for Gloucestershire, Herefordshire, Somersetshire, Staffordshire, Wiltshire, and Worcestershire, has compiled a similar return to that of Mr. Jenner Fust. In Gloucestershire the figures for the principal Unions are—Bristol, 62 paid nurses and 20 pauper attendants; Cheltenham, five paid nurses and nine pauper attendants; Gloucester, six paid nurses and no pauper attendants; Stroud, six paid nurses and four pauper attendants; Westbury-on-Severn, two paid nurses and six pauper attendants. Of the nurses in Gloucestershire thirty-four are fully trained. Herefordshire shows up rather badly, there being in the five unions of Bromyard, Dore, Hereford, Ledbury, and Ross, 11 paid nurses, five fully trained, and 13 pauper attendants. Somersetshire, on the other hand, has 19 paid nurses against two pauper attendants, and of the former ten are fully trained. In Staffordshire, the West Bromwich Union employ 38 pauper attendants and only eight paid nurses, of whom five are fully trained. Wolverhampton has 15 paid nurses, of whom four are fully trained, and eight pauper attendants; Walsall, ten paid nurses and four pauper attendants. The figures for Wiltshire indicate that there are seven paid nurses employed by the two Unions of Cricklade and Wootton Bassett, Swindon and Highworth, as against five pauper attendants; but of the former only two are fully trained. The most important of the Worcestershire Unions is Dudley. Here there are eight paid nurses, three fully trained, against 27 pauper attendants; Kidderminster has seven of the former and 12 of the latter; Stourbridge, six and 20. The Worcester, Bromsgrove, and Droitwich Unions have got rid of pauper attendants, Worcester employing five paid nurses, Bromsgrove three, and Droitwich two. The Worcester Union is in the van of progress, all of the five nurses being fully trained.

### DUBLIN NURSES' CLUB.

A meeting of the members of the Dublin Nurses' Club was held on Wednesday evening last week and was very well attended. The chief feature was the reading of essays by members of the club for which two prizes were offered by the directors of the City of Dublin Nursing Institution. The first prize was won by Miss Butler of Sir Patrick Dun's Hospital, who read an essay on "The Preparation of and Nursing a Case of Abdominal Section Throughout," and the other by Miss Young of the City of Dublin Nursing Institution, whose subject was "Manners." Both essays were much appreciated by those present, and an interesting discussion followed. It is hoped that during the next session an increasing number of the members will give the club the benefit of their studies.

### DAILY PAID VISITS AT BURTON.

At Burton-on-Trent there is, in connect on with the nursing institution, a branch of work called "The Daily Paid Visits." It is chiefly worked by the district nurses, the institution having both district and private nurses attached to it. According to the annual report these visits yielded £23 7s. They were 224 in number, and were, the report states, "paid to persons whose positions could not allow of their being district patients, but who were able and willing to pay a small fee for the services of a nurse." The danger of this arrangement is lest any of the time which the district nurses ought to be giving to the poor who cannot afford to pay, and for whose benefit they are at work, should be spent in tending the paying patients. But as in a further paragraph in the report stating that "the nurses have also earned £4 10s. in taking night duty," the words "when not required for district cases," are added. We assume that due care is observed to prevent the interests of the latter from suffering.

### THE NOTTINGHAM ASSOCIATION AND QUEEN VICTORIA'S JUBILEE INSTITUTE.

At the fifth annual meeting of the Nottinghamshire Nursing Association it was stated that during the year one local association, Collingham, had discontinued its work, but that two others, Coddington and Skegby, had started. Skegby is a colliery district and there is every prospect of the nurse's services being turned to good account. The association now employs 25 nurses, working among a population of close upon 100,000 people. But the feature of the meeting was the discussion on the proposal to affiliate the association with Queen Victoria's Jubilee Institute. It was admitted that increased expenditure would be necessary, but after an address from Miss Hughes, County Superintendent of the Institute, it was agreed by all the subsequent speakers that the advantages to be secured by the affiliation scheme were worth the additional outlay.

### A QUESTION OF TRAINING AT ARMAGH.

The Armagh Board of Guardians continue to get into hot water over the nursing question. Having to choose a nurse for the fever hospital in the workhouse, they avowedly selected one on the ground of her religion. The interest, however, centres in the fact that upon the appointment being notified to the Irish Local Government Board, the latter stated that as the nurse chosen, because she is a Protestant, could not produce a certificate of proficiency in compliance with the terms of the Nursing Order of July 5th, 1901, they were unable to sanction it. This proves that the Irish Local Government Board intend that the Order shall be obeyed in the spirit as well as in the letter; and it should have the effect not only of teaching Irish Boards of Guardians a lesson, but also of impressing upon nurses the uselessness of applying for posts unless they can produce certificates in conformity with the regulations.

### PLANS AND PROCEDURE.

If the Newton Abbot Guardians have no difficulty to overcome they create one. They lately decided to erect a nurses' home, which is very badly wanted, and the Building Committee prepared pencilled plans. These it was proposed to forward to the Local Government Board before the guardians had approved them. Reasonably enough, several guardians protested that they were entitled to learn the nature of the plans prior to the submission of them to the Local Government Board, and an animated discussion followed. Eventually, one of the protesting guardians declared that to bring up such a plan and expect the guardians to accept it before they had an opportunity of seeing what the committee wished to do, was a farce, and the further consideration of the matter was deferred for a fortnight. We hope that squabbles amongst the guardians will not delay the commencement of the home.

### ACTION AGAINST A NURSING INSTITUTE.

A case of some interest was heard in Conway County Court the other day. The plaintiff was Miss Ellen Boardman, a nurse, who sued the proprietor of the Llandudno Nursing Institute for a month's wages in lieu of notice. Miss Boardman had been wise enough to have a written agreement, and she had not lost it. This document proved that she had been engaged at a salary of £2 5s. a month, and was entitled to receive, as well as required to give, a month's notice. The defendant put in the plea that the nurse discharged herself, in which case he could already have obtained damages against her. But he did not set up a counter-claim, and the judge decided in the plaintiff's favour.

### PROGRESS AT SOUTHAMPTON.

The number of cases visited by the nurses attached to the Southampton Jubilee Nurses' Institute has now become so large—11,347 visits were paid in 1901—that a question has arisen as to the best course to be pursued. Either some limit of distance will have to be fixed, outside which the nurses must not be expected to be called to work, or the staff will have to be increased in number, as at present they are beginning to feel overtaxed. The latter would seem to be the best method, and the committee would be the more justified in such a proceeding because, whereas hitherto during most of the nine years of its existence the institute has suffered from financial difficulties, it has now reached less troubled waters. This is owing to the receipt of an anonymous gift of £1,000, which will produce £30 per annum, and to the generosity of Mr. Andrew Barlow, who has handed over to the society a mortgage producing £130 a year, less income tax. A vote of thanks has been given to the donor.

### MALE NURSES IN GENERAL HOSPITALS.

With reference to the article by a contributor who advocates the introduction of male nurses in general hospitals, there are, no doubt, cases which are not suitable for women to nurse. She herself cites some, and others could, of course, be added. At present the difficulty is to obtain a trained male nurse who is nearly as efficient as an ordinary probationer at the end of her second year. Moreover, in the present condition of the labour market the fact has to be faced that, while nursing attracts women of above the average type, it is likely to draw to itself men who wish to shirk harder work in other fields of employment, and this introduces many dangers. A case came under our notice a short time since. The man was very wealthy and very ill. His disease was not such as his friends thought a woman should attend, so they engaged a male nurse. For some weeks the patient made very little progress, and even the advice of a specialist produced no better result. It was ultimately discovered that this was almost entirely due to bad nursing. When the doctors were likely to come the nurse administered alcohol or drugs to unduly excite the patient in order that he might appear at his worst. In the evening, or when he wished to adjourn to the servants' hall or even occasionally to go to the theatre, the nurse gave sleeping draughts, cleverly tying the sufferer in bed under the sheets so that there might be no chance if he awoke of his ringing for assistance, or slipping out of bed. When these and many other irregularities were discovered, the man was discharged, and an ex-orderly was engaged, under whose care this time the medical directions were exactly carried out, the patient speedily became convalescent, and ultimately entirely recovered.

### A NURSES' CO-OPERATIVE SOCIETY AT JOHANNESBURG.

We have received particulars of a scheme which has been set on foot at Johannesburg to start a Nurses' Co-operative Society in that town on the same lines as the Nurses' Co-operation in London. Lord Milner is the patron; Lady Maxwell and Lady Tullibardine are the lady patronesses; and there is an advisory committee of six well-known ladies. The committee of the Transvaal Medical Society have expressed their sense of the importance of a central home and organisation for properly trained nurses at Johannesburg, their sympathy with the movement, and their readiness to employ the nurses and to inspect and express their opinion on the qualification of every nurse who desires to join. Miss J. E. Borlase, formerly of the Army Nursing Service Reserve, is acting as secretary, and will be the matron at "The Gables," Jeppe Street, which has been secured as a temporary home. We are informed that the nurses attached to the institution will take their own fees, less a small percentage, which will go towards the working expenses; and afterwards, when funds will allow, it is proposed to build a set of residential chambers which can be let to them at a fair rental.

### NEW HOME AT BARNSLEY.

The new Nurses' Home which has just been opened in connection with the Beckett Hospital and Dispensary at Barnsley is to be called the "Cooper" Home, after its donor. It is adjacent to the hospital building, and besides spacious entrance hall, dining-room, and matron's room, contains 18 comfortably furnished bedrooms, bath-rooms, etc. It is heated throughout, and is lighted by electricity. The result of Mr. Cooper's gift is that the committee are now able to provide a separate ward for the children, and otherwise to extend the work of the hospital.

### A YEAR'S INCOME IN HAND.

It is gratifying to learn that after three years' existence the Newry District Nursing Society is fully recognised as a useful and necessary institution. The annual report says: "It has established itself in popular favour, it is gratefully appreciated by the poor of our town, it has received generous and sympathetic support, and it is with much satisfaction that the committee report that the income for the past year has been in excess of the expenditure." Another pleasing feature is that the work of the society "has been greatly facilitated by the whole-hearted co-operation of the medical men," though this is the rule in such cases rather than the exception. When the association was formed it was decided not to start work until the committee had the year's income in hand, and this condition of financial stability has been observed ever since. The committee rightly insist upon the importance of maintaining it.

### Lectures on Gynæcology for Nurses.

By Robert Jardine, M.D., M.R.C.S., F.F.P. and S.G., F.R.S.E., Senior Physician to the Glasgow Maternity Hospital, Examiner in Midwifery to the University of Glasgow.

### IX.—VAGINITIS.

*Vaginitis.*—By this term we mean inflammation of the vagina. In the discharge different forms of micro-organisms can be demonstrated. The most important causes are: 1. Gonorrhœal infection, which may last for a long time and ultimately spread upwards into the uterus and Fallopian tubes. 2. Irritating uterine discharges, as from endometritis, cancer, etc. 3. Injuries, as from pessaries, vaginal injections, etc. 4. From exanthematous diseases, as scarlet fever, diphtheria, typhus, small-pox, etc.

*Symptoms.*—The patient complains of a burning pain in the passage, and she has frequency of micturition, accompanied with a scalding pain. In gonorrhœal cases the urinary symptoms are very pronounced, as the gonococci invade the urethra. There will be a free discharge from the vagina, and the mucous membrane will appear granular and red in patches. The presence of a vaginal discharge does not always mean that there is vaginitis, as the discharge may be coming from the uterus. The two often occur together. By means of a speculum it can soon be decided whether the discharge is coming from the uterus alone. The urinary symptoms are usually very acute in these cases, and treatment does not so quickly give relief.

*Treatment.*—In acute cases the patient must rest in bed and have sexual rest as well. The bowels will probably have to be freely moved by means of salines. Copious hot-water douches should be used several times daily. Morphia sup positories may be required if the pain is severe. For the urinary symptoms alkalies combined with belladonna or hyoscyamus are often given. When the acute stage is over, astringent antiseptic douches are useful, such as 1-3,000 corrosive sublimate, etc.; zinc sulphate, acetate of lead, etc., are also used. Various medicated pessaries are useful. Sometimes dry packing of the vagina after douching gives good results. Powdered boracic acid may be used in this way. In cases arising from endometritis, cancer, etc., vaginal douching will do good and give great relief to the patient, but to cure the condition the cause must be removed.

*Vaginismus.*—This is a painful reflex contraction of the muscular fibres surrounding the vaginal orifice. It is sometimes found in very nervous patients without any local source of irritation, but in most cases a cause can be found.

*Treatment.*—The cause must be sought for and removed. A very thorough dilatation of the vagina under anæsthesia will often cure the condition. The sphincter is forcibly stretched and paralysed for a time. In fissure of the anus, the anus is treated in the same way, and this allows the fissure to heal. Where the vagina is very narrow the patient may require to wear a dilator for some time.

*Urethral Caruncle.*—This is a small vascular growth which forms at the mouth of the urethra. It is bright red in colour and may be seen projecting like a piece of a strawberry from the meatus. It is very tender and will bleed easily. It will cause vaginismus and all its consequences, but it also causes great pain on micturition and when the patient is sitting. It is commoner in elderly than in young women. The diagnosis is made by inspecting the parts, when a bright red papilla will be seen projecting at the meatus urinarius.

*Treatment.*—It must be thoroughly removed, for which purpose the actual cautery or fuming nitric acid are sometimes used, or it may be snipped off and stitched. It is so tender that a local anæsthetic such as cocaine will be necessary.

*Injuries to the Vagina.*—The vagina may be injured by violence, as by a woman falling astride of a broken chair, or against the sharp edge of a tub, or by having something pushed into the passage. During delivery the vagina may be torn, or so bruised that sloughing takes place and an opening may form into the bladder, a vesico-vaginal fistula, or into the rectum, a recto-vaginal fistula. Both may form in the same case. Ulceration of the vagina may also be caused by ill-fitting pessaries, or pessaries which have been worn for a very long time without being removed and cleaned, for if a pessary is left in the vagina for many months it will become crusted over with phosphates and will cause great ulceration. Ulceration of the vagina sometimes occurs in fevers. It may also, be caused by syphilis and cancer. Occasionally very serious ulceration has been caused by a too hot douche or the application or a strong caustic. When the ulcers heal, especially those in which much tissue has been destroyed, there is certain to be a considerable amount of cicatricial contraction. This may be so great as to cause complete closure of the canal. We sometimes find cicatricial contractions obstructing the passage during labour.

*Treatment.*—Recent wounds of the vagina are treated in the same way as wounds in any part of the body. They are thoroughly cleansed and carefully stitched with catgut, any large bleeding vessels being tied in the usual way. If the woman is pregnant the bleeding may be very severe, as the parts are then very vascular. It may be necessary to plug the vagina, especially if there is much oozing. Daily douching will probably be necessary until the parts are healed. Tears which occur during delivery must be treated in the same way, viz., by stitching. Tearing of the posterior vaginal wall and perineum is a very common accident of delivery. If the tear extends into the rectum the parts must be very carefully cleaned and catgut stitches put into the tear in the bowel and tied inside the bowel. The perineal tear and the vaginal one must then be closed. Silkworm gut makes the best stitches for the perineum. When the bowels are moved care must be taken to get the motion as fluid as possible to prevent strain on the parts. An enema of warm olive oil is often useful. The bowels need not be disturbed for three or four days. The perineal stitches can be taken out in eight or ten days. Ulcers of the vagina must be kept as clean as possible. Foul ulcers are sometimes thoroughly swabbed with pure carbolic, care being taken not to allow the acid to affect the healthy mucous membrane. Iodoform powder mixed with boracic acid makes a good application. To prevent cicatricial contraction the vagina should be kept plugged. In syphilitic ulceration anti-syphilitic treatment will be necessary. Vesico-vaginal and recto-vaginal fistulæ we shall deal with more fully in our next lecture.

### To Nurses.

**We invite contributions from any of our readers, and shall be glad to pay for "Notes on News from the Nursing World," or for articles describing nursing experiences, or dealing with any nursing question from an original point of view. The minimum payment for contributions is 5s., but we welcome interesting contributions of a column, or a page, in length. It may be added that notices of appointments, entertainments, presentations, and deaths are not paid for, but that we are always glad to receive them. All rejected manuscripts are returned in due course, and all payments for manuscripts used are made as early as possible after the beginning of each quarter.**

### Private Nursing in South Africa.

By A TRAINED NURSE.

I AM so often asked for information as to the prospects of nurses coming out from home, that I have decided to give my opinion as plainly as possible to any nurses who will be glad of my experiences. I do not want to discourage any from coming out; indeed, we are all more than willing to welcome members of our profession who have made up their minds to live in sunny South Africa, and who have also before leaving home found out exactly what lies before them. And if in my desire to be honest I seem to "take the gilt off the gingerbread" somewhat, I must ask for forgiveness, and hope that the reality will be found more agreeable than my description.

### TRYING CLIMATE AND HARD WORK.

I cannot too strongly advise that those who are already tired out with the strain of private work at home, had far better remain where there is some comfort to be had. To be a successful worker in South Africa, one should be fairly young and more than fairly strong. The climate is especially trying to older women, and work of any kind more arduous than at home. Were it not too personal a matter I should like to give, for the benefit of other women, an account of the experiences of several nurse friends who have come out hoping to find work lighter and pay better than at home; these have generally been fortunate enough to find some other work than nursing and so have not been obliged to return to England. If people in this country realised the necessity of engaging two nurses for a serious case (the majority of cases are enteric or pneumonia) the way would be clearer, but this is not usually the case. It is difficult to obtain two nurses at once, except in towns like Capetown, and then, again, there is the extra expense, a much more serious matter to colonials than we at home imagine.

### SLEEPING UNDER DIFFICULTIES.

Now consider how much rest one can get taking the patient at night as a matter of course, remaining on duty to see the doctor at about 11 A.M., then, as is too often the case where the accommodation is limited, waiting until a bedroom has been prepared for one's reception. I have sometimes turned in at 11 A.M., and been asked if I would get up to dinner at 1, or have it brought to me! I have usually declined this offer, made in sheer ignorance, and in good faith, and done my best to get some sleep, while the children ran up and down the passages playing their games quite happily, and the Kaffir servants in the kitchen, probably next to your room, talked and laughed at the same time as only natives can. They verily "take no thought for the morrow" and very little for to-day. In these one-storeyed houses the walls are more or less partitions, and every sound echoes through the house.

### THE KAFFIR SERVANTS.

Again, the native servants in the Eastern Province, do not live in or even near the houses where they work. They live in a native "Location," generally some miles from the town. In some cases, and especially up country, the boys may be coaxed into occupying an outside room in the yard but this does not appeal to the Kaffir mind, they are so fond of "beanfeasts" and festive gatherings that you can never be sure when they are sleeping at your house or are taking the night off. But what has all this to do with private nursing? A very great deal, as you will soon discover. The Kaffirs leave their work at 5 P.M. or a little later, so that after that hour all domestic duties fall on the family, or in case of sickness, on the nurse, who, if she be worthy of that name, will gladly do her part. Another great drawback is that it is against the Kaffir notion of decency to empty slops or do anything of that kind; this, therefore, falls to the nurse's lot, unless some inferior, or to use their own word "rubbishy" native can be bribed into doing it. I am quite aware that these duties fall to the nurse at home, but things are differently managed here.

### PRIMITIVE SANITARY ARRANGEMENTS.

The sanitary arrangements are of the most primitive kind; that abomination, the night-cart, reigns supreme, except where, as in parts of Capetown, the water supply is plentiful and modern methods have been adopted. In nursing enteric, where one has to burn or bury the excreta, the usual plan is to dig a large hole or holes in the garden and there empty everything. You can imagine what this is, for in winter the cold is intense, and it is distinctly wearying to wander some 50 yards on a dark night carrying a pail. I have been using wet packs in an enteric case, and after placing a bucket of water in the yard at midnight, have found the water frozen over at 4 A.M., and my hands too numb to wring the cloths out. Very few bedrooms have fireplaces, and I have sat shivering with the cold though wearing a huge fur-lined cloak. All this sounds very gruesome, but to an English nurse who is strong and also keen on her work, it need not be discouraging. One meets with much kindness, and I think that socially a nurse has a better time than at home, but she must be prepared to put her hand to anything.

### THE QUESTION OF FEES.

The usual fee is £2 2s. per week, as at home; if a nurse has sole charge of a case, she generally asks for £3 3s., and her washing is mostly done at home with that of the family. The days (not long past) when trained nurses were so scarce that in Johannesburg many nurses received £6 6s. per week, are alas! no more. Board between cases can be obtained at about £6 per month, though since the war began and the prices of provisions have gone up, higher terms are asked.

### IF POSSIBLE, PAY YOUR OWN FARE.

As to the ways and means of getting out, that subject, though a very important one, does not come under the heading I have chosen. I strongly advise anyone who can do so to come out independently of any society, and I have rarely heard of a case where a nurse has given her services in lieu of travelling expenses being satisfactory. Probably she has undertaken the care of a tiresome child (most children are very tiresome on board ship, and when I see a child behaving well on board I always feel sure he is sickening for something or is too good for this world.) And not, perhaps, having been a voyage through the Tropics before, she finds herself *hors de combat* for at least part of the voyage. Mamma, who adores her son when she has very little to do with him, is not too well pleased with nurse, and is more than likely a bad sailor herself, and thus the situation becomes far from happy. Fares by some of the lines are very moderate, and two or three nurses joining forces could come out together very comfortably and cheaply, being careful to have some funds at their disposal on arrival. I must add that all nurses who take up private nursing should, if possible, have the L.O.S. certificate in addition to their three years' certificate. Indeed, there is plenty of room for nurses to practise midwifery alone, but they should be trained nurses as well.

### Wants and Workers.

MERLIN CHAIR wanted by very poor invalid nurse. Address Mrs. B. Farmer, Claremont House, Wimbledon Common.

ANY nurse who would like to possess the back numbers of THE HOSPITAL for the last seven years can have them, by paying the carriage, on application to Miss Wilson, 3 South View, Spring Road, Wrexham.

### "The Case Against Hospital Nurses."

A Criticism by Mary Gardner, Lady Superintendent, Birmingham and Midland Counties Sanatorium.

Is the editor of the *Nineteenth Century* influenced by any bias against the nursing profession? It seems impossible, yet a sweeping indictment appears in the April number entitled "The Case Against Hospital Nurses," this being the third attack of the kind within a comparatively short period in the same review. And it is hardly claiming too much to say that such attacks upon any professional body are rare in a non-professional journal, unprecedented, I think, in a leading monthly magazine.

The author, Miss M. F. Johnston, opens her case in this wise: "The nursing profession is curiously unpopular, and the feeling against it is steadily on the increase. . . . There are many people who dread to have a hospital nurse inside their doors, and who feel when disease invades the sacred precincts of the home, and when perhaps the shadow of death is dimly felt to be hovering near, that the situation would be shorn of many of its terrors if only it were possible for them to meet the exigencies of the case themselves and not be obliged to have recourse to professional assistance." The subsequent charges may be classed under three heads, coarseness, selfishness, and indifference, "an indifference amounting almost to brutality." This is not an over-statement of the accusation. These very words are used, and amplified in detail. The indictment, as the writer herself says, is a formidable one. By what evidence can it be supported?

Some statements require no proof, the truth is so obvious. Miss Johnston would seem to assume that her charges are of this kind, for she makes little or no attempt to substantiate her assertions, but rather takes the case for granted, appealing to general experience in confirmation thereof, and proceeds to investigate the cause or causes of this unhappy result. And her statement in this departure may be summarised in brief as follows:—That nurses are systematically overworked during their training, and that long hours, over-fatigue, lack of consideration on the part of those in authority, even consistent "snubbing and bullying" and every kind of petty tyranny from matrons and sisters (poor matrons and sisters!), result in jaded physique, indifference, want of sympathy, and a fatal narrowness, the least harmful effect of which is shown in the tendency to talk only "shop."

There are two assertions to deal with, viz. that the majority of nurses are, as a matter of fact, coarse, callous, and selfish, and that their hospital training and experience are such as inevitably tend to make them so, the general result of overwork and harsh treatment being, as is well known, to brutalise the subject. Can this be said with the faintest shadow of truth of hospital life? Miss Johnston admits that much has been done of late years for the welfare and comfort of nurses, and those who are familiar with hospitals know that in nearly all the staff has been greatly increased, in some instances doubled. This is not an undiluted gain, for the art of nursing is more elaborated to-day; nevertheless, the greater subdivision does make for a lessening of labour. If the life now be Egyptian slavery, too heavy to be borne, what must it have been in former days? Yet, under the older system, with all its hardships, was evolved a class of nurses of whom Florence Nightingale, Agnes Jones, and Sister Dora were conspicuous and shining examples. But they were conspicuous by accident of circumstance, not by character. We may claim them as types, not rarities. I have known many nurses who in character and devotion might fitly be ranked with the bearers of those honoured names. Be it observed that I am not defending the old system, which was too rigorous and has been gradually reformed, but am merely speaking of its effect upon character.

And what of the modern system? So far as hospital nurses to-day are concerned to whom can we appeal? Surely to the patients. Let anyone who wishes to find out the truth of this matter, visit any of our large hospitals, watch the nurses with their patients, question the former as to their happiness or otherwise in their work, and the latter as to the care and attention they receive. Or question the patients outside, after they have left the hospital, and judge by their testimony. At the risk of seeming egotistical I must speak of myself so far as is necessary to justify my claim to give evidence. As a nurse, yet standing outside hospital work, I may be a more impartial witness than some. I will not draw upon recollections of my own nursing days, but speak of the present. I am in charge of a large convalescent home where we receive patients from many hospitals, and their testimony is unvarying as to the care and kindness received from the nurses. They are glad to leave the hospitals, being weary of the confinement and monotony of sickness, but they part often with keen regret from those who have attended them. If one hears a complaint it is of having to lie in bed so long, or perchance of the restricted diet, but never of the attention shown, unless, indeed, one meets with an inveterate grumbler, such as would not be happy without a grievance in heaven.

I will venture to challenge Miss Johnston's accusation by asserting that the "case against hospital nurses" is not only unproved, but by overwhelming testimony may be proved to be grotesquely untrue. I do not say wilfully or maliciously so. I have dealt with the accusation so far only as it touches hospital nurses. The framing of the title makes it apply to such, but the charge as set forth in the article is really directed more against private nurses, and on this ground it is more difficult to meet. But there is one test which works automatically, *i.e.* the law of demand and supply. The demand for private nurses has unquestionably increased, and often exceeds the supply. Would this be the case if they were really so bad as represented?

Unfortunately, in so large and heterogeneous a society there will be found some black and grey sheep, more perhaps in the ranks of private nurses, because they are more uncontrolled than in either of the other branches of nursing. But do those who criticise and condemn so unsparingly the private nurse, ever think of what she has often to put up with in her turn, of the "callousness, selfishness, and indifference" which she encounters, often the result of pure ignorance, of want of thought rather than want of heart, as exemplified in the common delusion that nurses can do without sleep?

"I could a tale unfold" on this head, but it is not worth while. Retort is neither argument nor justification. I will only add that having been hospital, district, and private nurse I say unhesitatingly that the private nurse has the hardest life, the most wearing and exhausting in the long run, though she has, as Miss Johnston says, but "one patient to attend to." That shallow view of the outsider!

These hysterical outbursts against the martyrdom or the depravity of nurses are becoming tiresome. Yet even an extravagant accusation may be of service as a warning that a prejudice exists against nurses in certain quarters, and lead them first to look at home and see that their individual conduct is worthy of their high tradition, and secondly to do each one her part in purifying the ranks. Hospital matrons have great power in this direction, but every nurse can do something, directly or indirectly. And, finally, we may rest assured that "If this counsel or this work be of men it will come to nought. But if it be of God ye cannot overthrow it." Does this seem to anyone profane? Yet it is not so, for the highest examples and precepts give the law to the common things of life.

### A Visit to a Deaconesses' Hospital in the Black Forest.

BY A CORRESPONDENT

Whoever is lucky enough to find herself in that lovely corner of Germany, the Black Forest, will probably finish up her wanderings by paying a visit to Freiburg, that quaint old town boasting a university and a minster whose tapering spire shows like fretwork against the sky. But while proud of its mediæval buildings old Freiburg marches with the times, and I should advise anyone interested in hospitals to obtain the entry to one which has been so successful that, although not more than three or four years old, it is already proving too small for the demands made upon it.

### The General Arrangements.

A short time ago, as I approached it through softly falling snow, I could not but admire its picturesque appearance, the whitened turrets, gables, and dormer windows reminding me of many an old German castle, while various modifications showed it to be a very modern hospital. All the wards and a long verandah face south and look out upon large gardens, where the convalescent patients can wander in summer breathing in the fresh air from the pine-covered mountains. After passing the door leading into the chapel I reached the chief entrance, and, after introducing myself as a nurse on her travels, was warmly welcomed by the matron, or "house mother," the pleasant title so often used in Germany. The hospital contains 88 beds, including 19 first-class, 13 second-class, and 56 third-class patients, paying respectively 6s. to 9s., 4s. to 5s., and 2s. 2½d. a day. In the spacious basement are the dispensary, the sterilising and disinfecting apparatus and boilers supplying hot water to every part of the building. On the ground floor the matron, doctors, and chaplain have their quarters, and there are dining and sitting rooms for the nurses, as well as the kitchen and offices. The first floor is devoted to surgical cases; there are 47 beds. The first and second class patients occupy several small nicely furnished rooms, and there are not generally more than five or six cases in a third-class ward. A good bath-room, operating theatre, ward kitchen, and store-rooms complete this department. On the floor above are the medical wards, with 37 beds in 19 rooms, fully provided with vapour, hot air and other baths, and apparatus for gymnastic and electrical treatment.

### The Accommodation for Nurses.

Originally the top storey was intended exclusively for the nursing staff, but owing to the pressure of cases some of the rooms have been taken for patients, the result being that the accommodation for nurses is very limited, not even the sisters having separate bedrooms. The matron told me that, owing to this state of affairs, the question of building a nurses' home was being considered. Apart from the main building on the north side are the laboratory and dissecting rooms, the laundry, and a little isolation hospital containing four beds. In speaking of the nursing of this hospital, it must be remembered that it is carried on under the rule of Evangelical deaconesses, but many of the nurses never take the last step of wholly dedicating themselves to life-long service amongst the sick. Perfect freedom is allowed, and as the training takes several years a nurse has plenty of time in which to discover whether she is fit to be set apart to lead the active religious life of a consecrated deaconess.

### The Staff.

The staff consists of nine fully-trained sisters and deaconesses with 30 probationers, a dispenser, and a schoolmistress; but so great is the demand for district nurses amongst the sick poor—there are at present 44 employed in Baden belonging to this hospital—that the matron told me she had difficulty in keeping enough experienced sisters to train the probationers. At first sight the staff seems excessive, but no wardmaids are kept, and many of the probationers are employed in cooking, washing, sewing, etc.; only 12 are actually nursing in the wards. Owing to the demand for nurses at present greatly exceeding the supply girls of 16 or 17 are taken, quite too young for general nursing. Great care is exercised to admit only very healthy girls, and as many come from farms where they are accustomed to a hardy life and hard work, they do not break down so frequently as our English nurses, who are generally drawn from a higher and less robust rank.

### Three Grades.

A candidate has to produce eight certificates of health, character, etc. (everything is classified and registered in Germany) and if accepted she is on trial for two months or longer. Then if proved satisfactory she is given a cap marking her as a probationer or "Lehr-Schwester," and now she has to mount three grades, according to her capacity reaching the top quickly or slowly; but in only very exceptional cases can a probationer reach the third class before she is 20. As a rule the first class contains all nurses under that age and others who still need training in cleaning, cooking, sewing, knitting, darning, washing, ironing, and gardening! Village nurses especially are expected to thoroughly understand all branches of domestic economy. In the second class general instruction is given when necessary, and probationers are taught book-keeping, letter-writing, theoretical nursing, as well as singing and such elementary natural science as may help them in their profession. In the third class the probationers are employed entirely in nursing, either in the hospital or in the homes of the poor.

### Salaries and Allowances.

At the end of each year probationers receive uniform, £1 15s. for shoes, a month's holiday, travelling expenses, and £3 per annum pocket money, which after the age of 20 is increased to £5. After 10 years' service a deaconess gets £7 10s. So it is evident that it is not a high salary which induces women to join this branch of the profession. On the other hand, every deaconess has the comfort of knowing that when overtaken by sickness or old age her Mother house stands with open doors ready to receive her; or if she prefers living with her own friends she is assured a pension of £20 a year for life.

### Novelties for Nurses.

By Our Shopping Correspondent.

### CRADLE SONGS.

The Frame Food Company have published a nice little collection of lullabies and cradle songs, which they will be pleased to send to any of the readers of The Hospital, who send a postcard asking for it, addressed "Frame Food Company, Battersea, S.W."

### FOR THE WARDROBE.

Messrs. Grossmith and Son, of Newgate Street, are well-known perfumers, whose specialities may be procured wherever scents and toilette accessories are sold. Their sachets and sachet powders are especially to be recommended, and the sachet perfumed with Phul-nana is the nicest of all. It has the most lasting properties, and is delightful to introduce amongst handkerchiefs.

### ORLWOOLA.

I have received from Messrs. Gorringe some pretty patterns of the "unshrinkable pure wool flannel" known as Orlwoola; they are in plain colours and checks, and the colours range from light to dark, in pinks and reds, greens and blues, while a sombre black and white would answer the purpose of half-mourning excellently. For blouses, dressing gowns, and underwear this material is very satisfactory. The width of the patterns sent me is 31 inches; the price is under two shillings a yard.

### Male Nurses in General Hospitals.

BY AN OCCASIONAL CONTRIBUTOR.

The war having familiarised us with the idea of the hospital orderly, one begins to wonder if that functionary, or someone resembling him, might not be found useful in civil hospitals. We all like the idea of the ministering angel nurse, but as a matter of fact, we know that nursing is such hard work in some cases that it tries too severely the strength of the nurse, who is also a woman with the limitations of her sex. Certainly the lifting of a heavy patient is a matter of skill as much as strength, and often a skilful woman will manage to move a helpless patient without apparently great effort, where an unskilled man would puff and pant, and probably cause the patient pain. But that is no reason for ignoring the man's greater strength, but rather for training it as the woman's lesser power has been trained. That some nurses suffer from the strain of lifting heavy patients we know, and it might be well that in the male wards of our ordinary hospitals there should be men nurses to help in such tasks.

Refractory Patients.

For other reasons, also, the presence of a man or two in the wards might be desirable. The class from which the bulk of hospital patients are drawn is not one that respects its womenkind. No doubt they may get a valuable lesson in courtesy and gratitude in the wards, but they are not always of a nature to profit by it. A good many nurses can tell tales of refractory patients, who were rude, troublesome, and perhaps abusive, feeling themselves safe from punishment by reason of their weakness, and also because they had only women to deal with. Had there been a man about to warn them that no nonsense would be tolerated, their behaviour might have improved. A nurse once told me of a stormy-tempered patient who bullied everyone in the ward except the charge nurse. When she was away on holiday he established a tyranny over nurses and fellow-patients alike. When the charge nurse returned, he greeted her with: "You devil, have you come back?" A few days later, while the nurse was busy at the end of the ward, she heard an exclamation of terror from someone behind her, and when she turned, saw this patient with his arm raised to strike her. When he saw that his attempt had been defeated he slunk away to bed, but he might, if he had not been intercepted, have inflicted considerable injury on the nurse for whom he had so great a dislike. On such a patient the presence of a male nurse would have acted as a restraint.

A Scandalous Case.

Occasionally patients are received into hospital which it is unfair to ask women to deal with. No one can be blamed for this. If the seriousness of a man's ailment justifies his admission there is no question asked as to his moral character. It is true that even a bad man, when he is ill and is tended kindly by good women will often, perhaps usually, behave with decency, and may even learn in the hospital wards a higher notion of virtue, but there are exceptions. For example, a man was recently received into a large hospital suffering from alcoholic paralysis, which however affected only his tongue. Perhaps there was about him a touch of insanity, induced by alcoholism, but as the case was in all probability curable, it was not strange that a doctor should admit him to a general hospital instead of sending him to an asylum, which, like the gaol, leaves a stigma upon those who have been within its walls which stands in their way in after life. Now this man, whether mad or merely bad, behaved in a scandalous manner. If he saw a nurse go into one of the kitchens at the end of the ward, he would jump up and rush after her. Most of the nurses were terrified at him. He had no sense of decency. He jumped out of bed and passed all evacuations on the floor, and would then lie and laugh when he saw the nurses wiping them away. Finally the charge nurse complained and a man was sent to attend to him. But for such a case should not the male nurse have been a matter of course?

Private Work.

It may be asked if the life of a nurse offers sufficient inducement to men to supply enough male probationers for the wards of our hospitals. There is always a demand for male attendants for invalid gentlemen, though perhaps this is not a large one, and in many cases little more than a valet is needed. Yet if a larger number of competent male nurses were available a greater demand for their services would arise, just as it has done in the case of women nurses. And in ordinary private work, just as in hospitals, there are cases which might preferably be nursed by men. One would certainly recommend a male nurse in a case like this, regarding which the following message was sent to a nursing home: "Please send a nurse; an elderly and sedate one, as the patient is a young man of twenty-five." Perhaps I wrong the young man, but I cannot help thinking that there were special reasons why his friends desired that his nurse should be sedate and elderly. It might not be practicable in many towns to have an institution devoted entirely to male nurses, but if ordinary nursing institutions had a few male nurses on their staff—they need not reside in the home—I believe, their services would gradually be more and more accepted and all the more if it were permitted to a woman nurse to refuse to attend such cases. Perhaps in time we shall come to think that even the largest charity and the most religious estimate of the nurse's calling does not compel us to send pure women to nurse vicious men in the diseases which are the direct result of their vices.

### Appointments.

Aston Union Workhouse Infirmary.—Miss Janetta Julia Lersey has been appointed home sister. She was trained at Puddington Infirmary, and has since done private nursing, and been charge nurse at St. Saviour's Hospital, Osnaburgh Street, London.

Borough Isolation Hospital, Darwen.—Miss F. K. Monkhouse has been appointed matron. She was trained for four years at St. George's Hospital, London, and has since held the posts of charge nurse at the South-Eastern Hospital, New Cross, ward sister at Southwark Infirmary, East Dulwich Grove, and home sister at St. Leonard's Infirmary, Shoreditch.

Devonport Workhouse Infirmary.—Miss Margaret Priestman has been appointed superintendent nurse. She was trained at the Mill Road Infirmary, Liverpool, where she afterwards held the post of sister for three and a half years. She has since been ward sister, and for a short time night sister at the New Isolation Hospital, Wimbledon.

High Peak Isolation Hospital.—Miss Emma Newton has been appointed superintendent nurse, and Miss Gertrude Jinks staff nurse. Miss Newton was trained at Ashton Union Workhouse Infirmary, and has since been charge nurse at Ashton-under-Lyne Workhouse Isolation Hospital. Miss Jinks was trained at the Union Infirmary, Fir Vale, Sheffield.

Louth Hospital and Dispensary.—Miss Maud Crichton has been appointed matron. She was trained for three years at Leeds General Infirmary, where she was afterwards sister for two years. She has since been night superintendent at the Hull Royal Infirmary.

Stirling District Asylum, Larbert.—Miss Susan H. Kerr has been appointed assistant matron, and Miss Helen S. Dykes night superintendent assistant matron. Miss Kerr was trained at Charing Cross Hospital, London, was afterwards sister for four years at the Royal Hospital, Portsmouth, and for two years sister of the ophthalmic wards and night superintendent sister, alternately, at the Greek Hospital, Alexandria, Egypt. Miss Dykes was trained at the Western Infirmary, Glasgow, and was for five years attached to the Glasgow and West of Scotland Co-operation for Trained Nurses.

TAUNTON AND SOMERSET HOSPITAL.—Miss Edith H. Grime has been appointed assistant matron. She was trained at the Royal Infirmary, Manchester, where she was staff nurse for 18 months, and has since been head nurse at Hertford General Infirmary.

VICTORIA COTTAGE HOSPITAL, KINGTON.—Miss May C. Robertson has been appointed matron. She was trained at the Children's Hospital, Gloucester, and Guy's Hospital, London, and has since been assistant nurse at the Cottage Hospital, Wellingborough.

WAKEFIELD UNION INFIRMARY.—Miss Fanny Lloyd has been appointed sister. She was trained at Toxteth Park Infirmary, Liverpool, and has since been charge nurse of the scarlet fever ward at Western Hospital, Fulham, London.

### Everybody's Opinion.

[Correspondence on all subjects is invited, but we cannot in any way be responsible for the opinions expressed by our correspondents. No communication can be entertained if the name and address of the correspondent are not given as a guarantee of good faith, but not necessarily for publication. All correspondents should write on one side of the paper only.]

### X-RAYS.

"NURSE G. S. B." writes: May I be allowed to answer a question asked in Notes and Queries by "X. R." in THE HOSPITAL of April 5th, and say that face and other parts not required to be exposed to X-rays should be protected by a double layer of lead-foil (1lb. to the sheet)?

### A SCHEME FOR AFFILIATED HOSPITAL NURSING.

"A PROVINCIAL MATRON" writes: As one who has had several years' experience as superintendent of a small country hospital I was much surprised on reading a "Former Matron's" article. I never had any difficulty in finding probationers. If by any chance my list ran short an advertisement produced 60 to 80 answers. The necessary age was 23 to 30, and the only "bait" offered was a certificate after three years' work. Whether these probationers would have been accepted on leaving in large London hospitals I cannot say, but as they had no difficulty in getting posts, either as charge nurses, district nurses, or on the staff of any private nursing institution they wished to join, they never tried. I consider 23 the very lowest age at which a woman should enter an adult hospital ward, and cannot see how the fact of its being in the country can make any difference.

### SLEEPING IN A PATIENT'S ROOM.

"HARDWORKING" writes: "Inexperienced" may have a great deal to learn if she needs to write for public opinion as to whether it is the proper thing for a nurse to sleep in a male patient's room, but "the more experienced private nurses" evidently require to learn even more: if they are so easily shocked at what to my idea is a nurse's duty in a helpless or bad case. If the patient only required "an occasional drink" it seemed quite unnecessary for a nurse to be at hand during the night; a covered-over drink, a night-light, and a hand-bell would in that case have been sufficient. For years I have done surgical work, and I think that in nearly every case—if I was the only nurse and there was no room leading out—I have either slept, after an operation, on the sofa or in a chair bed in the patient's room, and I must honestly confess that I feel none the worse for so doing. Directly the patient is able to help himself and all danger is past I go to my own room to sleep.

"AN OLD EXPERIENCED NURSE" writes: I am surprised to read in this week's HOSPITAL of the more experienced nurses in an Institute being shocked at "Inexperienced" sleeping in her patient's room, and I should like to know what they suggest doing when only one nurse is in charge. I consider that the nurse who has her work at heart and is jealous of every moment spent out of her patient's room is the only one suitable for private nursing. My own experience where one has sole charge is that it is much better to sleep in the room than run any risks. I often find more harm done during my off-duty than the rest has done me good. I have just left a typhoid pneumonia case, an elderly gentleman, and I slept in the same room six weeks, taking four hours off between poulticing for rest, and two hours at another time for exercise. I think the experienced nurses who would not dream of doing such a thing cannot be very conscientious nice women to suggest such thoughts to an inexperienced girl. I always find gentlemen patients appreciate a nurse without any false modesty.

### FEMALE NURSES FOR MALE LUNATICS.

"GEORGE M. ROBERTSON, MEDICAL SUPERINTENDENT," The Copse, Labert, N.B., writes: In this week's number of THE HOSPITAL there is an article on "Female Nurses for Male Lunatics" which takes no cognisance of what has been done at the Stirling District Asylum at Larbert. Nearly one-half the staff on the male side, by day and by night, consist of women. There is no head male attendant, but a matron of the male side, who is a trained hospital nurse, and there is no chief night watch, but a night superintendent, who is also a trained hospital nurse. The system has been in full operation for two years, and has proved not only feasible but a very great success. There have been no accidents and no scandal; the patients are less excitable and more amenable to order. The sick and the infirm and the acutely ill have been treated as tenderly as if they were in a general hospital. The account of these facts, as well as other innovations of a *hospital character*, such as efficient night nursing, the abolition of solitary confinement, and the employment of fully-trained nurses in the wards, appears in the April number of the *Journal of Mental Science*.

### PAUPER "NURSES" IN LANCASHIRE WORKHOUSE INFIRMARIES.

"MARY E. BALL, superintendent nurse at the Union Hospital, Jericho, Bury," writes: In your issue of March 29th, I was surprised to see some comments on pauper nurses in Lancashire Workhouses, and a statement that the condition of affairs in the Bury Workhouse, with two others, was absolutely scandalous. I know nothing of the others, but must take exception to the remark in regard to Bury. The paupers given as assisting in the sick wards are not assistant nurses. They clean, carry food, carry from laundry, etc. I know two workhouses in which widows receiving outdoor relief come in to clean, but they are not called nurses, neither are the wardmaids in a general hospital. I have before me, as I write, the Local Government Board's return for 1900, and find that then I was the only nurse here with a three years' certificate of efficiency. Now I have two three years' certificated nurses with others who have more or less experience in nursing. So much for reform. Mine is from the Brownlow Hill Infirmary, Liverpool, which you say is one of the best in the country. I also have the L.O.S. I have been a nurse for more than 23 years, have lived in several workhouses, and was sorry to see such misleading statements in your paper, as I think it very discouraging to a Board of Guardians who are always anxious to appoint the best candidates for posts in their hospital, and are now busy with plans for a new infirmary.

[The figures referred to are not ours, but those of the District Inspector of the Local Government Board, and represent, "the number of paupers (other than convalescents) assisting in the sick wards." It was not suggested that the superintendent nurse at Bury Workhouse Infirmary was not properly trained, nor that matters had not improved.—ED. THE HOSPITAL.]

### "The Hospital" Convalescent Fund.

THE hon. secretary acknowledges, with many thanks, the receipt of postal order for 5s., from Miss L. B. Marsh, Bishop's Stortford.

### Echoes from the Outside World.

### Movements of Royalty.

The South of England has been much favoured by visits from Royal personages of late and this week even the remote Scilly Isles have also been included. At the end of last week the King landed from his yacht and went over Portland Prison and the stone works, intending to proceed to Falmouth, but the winds were contrary and the vessel had to put into Plymouth. On Monday afternoon it arrived at St. Mary's, and subsequently His Majesty visited the Governor of the islands at Tresco, showing special interest in the flower farms which just now are at their best. The inhabitants, most of whom have never seen the King, even when he was Duke of Cornwall and consequently their landlord, were of course highly delighted at the honour paid to them, although the resources of the islands were very heavily taxed to procure flags and bunting. The visit was so unexpected that there was no possibility of sending to the mainland for supplies. On Tuesday the King spent some time at St. Mary's.

Queen Alexandra's father, King Christian IX. of Denmark, celebrated his eighty-fourth birthday on Tuesday. Copenhagen was decorated with English, Danish and Russian flags. A great many of his descendants assembled to do honour to the occasion, in addition to Queen Alexandra and the Prince and Princess of Wales. The King was one of ten children, of whom one died at the age of 21. Of the rest, four are now living at ages varying between 77 and 91. He ascended the throne of Denmark in 1860, and in 1892 the King and Queen celebrated their Golden Wedding. Queen Louise died in 1898.

### South Africa.

The amount of money left by Mr. Cecil Rhodes is about six millions sterling. Amongst his executors and trustees, of whom there are seven, are Lord Rosebery, Lord Grey, Lord Milner, and Dr. Jameson. The first clauses of the will relate to the place of burial. Mr. Rhodes selected the hill in the Matoppos, which he called "The View of the World." There the monument to those who fell in the first Matabele war is to be erected, and the burial place and its surroundings are to be yearly beautified out of funds left for the purpose; and there, too, either men or women, who shall, according to the votes of the Future Federal Government of South Africa, be considered to have "deserved well of their country," may be buried. A short railway is to be built from Bulawayo to the cemetery, and a public park is to be formed by the town. £2,000 a year is set apart for an agricultural college to train the people of Rhodesia in the best agricultural methods. £100,000 free of duty is bequeathed to Oriel College, Oxford, where Mr. Rhodes graduated. His beautiful home, near Capetown, is given, together with an endowment of £1,000 a year to the future Premiers of Federated South Africa. Sixty scholarships, each of the value of £300 a year for three years, tenable at the University of Oxford, are provided for by the will. All the principal colonies are mentioned, including the Bermudas and Jamaica. Neither race nor religious opinion is to disqualify, and fondness for sport, manhood, "moral force and instincts to lead" are to count among the necessary qualifications. Two scholarships of the same value as those for the Colonies are appropriated to each of the American States, of which there are in all 52. Because Mr. Rhodes believed "that educational relations form the strongest tie, and that a good understanding between England, Germany, and the United States will secure the peace of the world," he has left money for fifteen scholarships for Germans, tenable at Oxford, the nomination being in the hands of the German Emperor.

The funeral of Mr. Rhodes has provoked remarkable demonstrations of public feeling in South Africa. At Cape Town all business was suspended on the arrival of the body, which was laid in state at the Houses of Parliament. A wreath from Queen Alexandra was among those placed on the coffin. At the Cathedral, which was thronged in every part, the opening sentences of the Burial Service were read, and then the Archbishop of Cape Town delivered an address in which he dwelt on the charity and large-heartedness displayed by Mr. Rhodes. His Grace said: "Though not a great church-goer, Mr. Rhodes was essentially religious. In sacred converse with the Archbishop shortly before his last illness, he showed that he realised the nearness of death, and manifested an earnest desire to prepare to meet it."

On Monday evening the Secretary of State for War announced that he had received a telegram from Lord Kitchener, dated April 6th, stating that Commandant Kritzinger's trial had been concluded and the prisoner acquitted. Kritzinger, who will consequently be treated as an ordinary prisoner of war, is a native of Zastron, in Orange River Colony. He served under De Wet, and late in 1901 crossed the Orange and began a raid through Cape Colony which ended in his capture. He was charged with murder and other violations of the rules of war.

### Politics and Society.

The death of the Earl of Kimberley occurred on Tuesday. Lord Kimberley had been severely ill for many months, and the end was not unexpected. During his illness the King and the Prince of Wales were kept informed of his condition, and there were many anxious inquiries from all sorts of people. The deceased statesman, who was born in 1826, and never sat in the House of Commons, had a distinguished career, and was Under-Secretary for Foreign Affairs so far back as 1852. He was all his life a Liberal, and held in succession at intervals the posts of Under-Secretary for India, Lord Lieutenant of Ireland, Lord Privy Seal, Colonial Secretary, Secretary of State for India, and Foreign Secretary, finally retiring from office in June 1896.

### Catastrophe at a Football Match.

On Saturday a terrible disaster occurred at Glasgow, involving the loss of more than a score of lives and severe injuries to upwards of 300 persons. The scene of the accident was Ibrox Park, which was laid out at a cost of £20,000 two years ago, and was supposed to be one of the most perfectly equipped athletic arenas in the country. It has a covered grand stand at each side, and inclined terraces at each end open to the sky. All these were crowded on Saturday by spectators who had assembled to watch the International Association football match between England and Scotland, and one of the stands, with 33,000 people on it, suddenly collapsed, many hundreds falling to the ground about 40 feet below. For a time, instead of play, a procession of bearers crossing the field carrying the dead and dying into the pavilion, was seen. Many doctors were among the onlookers and lent ready aid, improvising stretchers and splints from the shattered woodwork. After an interval of 20 minutes play was resumed, and the work of rendering first aid to the injured and tending the dying went on amid the incongruous sounds of the cheering of partisans on the terraces. The King sent a message of sympathy from the Scilly Isles to the Lord Provost of Glasgow on Tuesday.

### The Drama.

The much talked-of "Ben-Hur" has been produced at Drury Lane, and bids fair to be as big a success in England as it was in America. The religious element introduced is very slight, and is treated in a thoroughly reverent manner. It consists of a tableau-prelude, the meeting of the Wise Men in the desert, and of the healing of two lepers at the end of the play. The actual deed is not seen, only the waving of palms and the sound of "Hosanna" show the nearness of the Saviour. The rest of the story deals with the fortunes and misfortunes of the young Jewish prince, Ben-Hur, most forcibly acted by Mr. Robert Taber. Mr. J. E. Dodson as a steward, Mr. Basil Gill as Ben-Hur's enemy, Messala, Miss Constance Collier as a beautiful alluring Egyptian, and Miss Nora Kerin as a sweet Jewish maiden, all deserve special praise, though the entire company is excellent. The scenery and costumes are magnificent, and the race in the hippodrome, where four chariots with teams of four horses race at their utmost speed, is bewilderingly exciting and realistic.

### For Reading to the Sick.

SPRING'S ADVENT.

Once more thou comest, O delicious Spring!
And as thy light and gentle footsteps tread
Among earth's glories, desolate and dead,
Breathest revival over every thing.
Thy genial spirit is abroad to bring
The cold and faded into life and bloom,
Emblem of that which shall unlock the tomb,
And take away the fell destroyer's sting.
Therefore thou hast the warmer welcoming
For Nature speaks not of herself alone,
But in her Resurrection tells our own.
As from its grave comes forth the buried grain,
So man's frail body, in corruption sown,
In incorruption shall be raised again.

*William Croswell.*

While we are still here the language of worship seems far, and yet lies very nigh; for what better note can our frail tongues lisp than the voice of wind and sea, river and stream, those grateful servants giving all and asking nothing, the soft whisper of snow and rain eager to replenish, or the thunder proclaiming a majesty too great for utterance? Here, too, stands the angel with the censer, gathering up the fragments of teeming earth and forest-tree, of flower and fruit, and sweetly pungent herb distilled by sun and rain for joyful use. Here, too, come acolytes lighting the dark with tapers—sun, moon, and stars—gifts of the Lord that His sanctuary may stand ever served.—*M. Fairlees.*

There are days in early spring when the flowers are opening, and the unfolding woods are sounding with song; the sky is softest azure, and the sun is bright; the fair undulating hills lie in dim blue outline, and the valleys are half veiled in hazy melting mist; all the earth seems dreaming, half wakening, and scarcely willing to be wakened, from its winter sleep. Another day the flowers still are opening, and the leaves are green; the sun is shining, and the skies are blue; but the woods are almost voiceless, and the distances are clear and hard, for a chill March wind is blowing, and nature is shivering, as she passes from her slumbers. Perhaps it is a whisper of the winter not quite dead. Perhaps it is a helpful wind, checking herbage too rapid to be rich. But, whatever it be, the warmer days are coming, and, cold or genial, it is the spring.—*Little.*

A NEW OLD SONG.

The Spring comes slowly up this way,
Slowly, slowly!
A little nearer every day.

In kirtle all of green and grey;
Slowly, slowly,
The Spring comes slowly up this way!

She hath delicious things to say,
But will not answer yea or nay,
Nor haste her secrets to display.

The pink is on the orchard spray,
The lambs put off their fears and play,
Gone are the snows of yesterday.

*K. Tynan.*

### Notes and Queries.

**The Editor is always willing to answer in this column, without any fee, all reasonable questions, as soon as possible.**

**But the following rules must be carefully observed:—**

**1. Every communication must be accompanied by the name and address of the writer.**

**2. The question must always bear upon nursing, directly or indirectly.**

**If an answer is required by letter a fee of half-a-crown must be enclosed with the note containing the inquiry, and we cannot undertake to forward letters addressed to correspondents making inquiries. It is therefore requested that our readers will not enclose either a stamp or a stamped envelope.**

*Pension.*

(12) I have worked as a nurse since 1879. Do you think that there is any chance of my getting a pension? If you could give me any idea I should esteem it a great favour.—*L. C.*

You are not eligible for a pension (excepting under the Poor Law Superannuation Act) unless you have subscribed to the Royal National Pension Fund for Nurses. You may, however, have a slight chance of an annuity from the Trained Nurses' Annuity Fund, 72 Cheapside, E.C.

*Small-Pox Nurse.*

(13) Do you think I might volunteer as small-pox nurse? I have no training except in maternity, but I have had small-pox myself.—*M. F.*

It would be quite useless for you to volunteer in the circumstances.

*Netley.*

(14) I am anxious to get into Netley. Will you kindly tell me to whom I ought to apply? I have nearly completed my training here.—*Charge.*

For admission into the Military Nursing Service you must apply to the Under-Secretary of State, War Office, Pall Mall, S.W.

*Varicose Veins.*

(15) Will you kindly tell me if there is any cure or relief for varicose veins of six years' standing?—*E. W.*

Yes. You must consult a good surgeon. If you cannot afford a specialist, go to the largest hospital in your neighbourhood. The General Hospital, Birmingham, seems the best for you.

*Taking Temperatures.*

(16) Will you kindly tell me where I could obtain a few lessons in taking temperatures, and in filling in charts?—*A. E. C.*

The Secretary, the Trained Nurses' Club, 12 Buckingham Street, Strand, W.C., would be able to help you.

*Lady Roberts' Nurses.*

(17) Can you kindly let me know if Lady Roberts' Nursing Association is still in existence? and to whom I should apply for the necessary papers, etc.—*J. H.*

Lady Roberts' Nurses are selected privately, and no outside applications are entertained.

*Antiseptics.*

(18) Is there anything in soap that would counteract the antiseptic effect of bui-iodide lotion? If so, is there any antiseptic that could be used with soap?—*Nurse Elizabeth.*

Soap itself is an excellent antiseptic. It is usual to cleanse the skin thoroughly with soap and water first, and then to apply the antiseptic lotion desired. There are a number of soaps into the composition of which an antiseptic has been introduced in the market.

*Port-wine Stain.*

(19) I should be glad to know if there is any treatment for the cure of a "port-wine stain" on a baby's face? He was not born with it; it has gradually developed. Mother would take the infant as out-patient to any hospital in London, if she knew which was the best.—*District Nurse.*

Everything depends upon the size and vascularity of the nævus. The case, therefore, is eminently one for expert surgical advice.

**Standard Books of Reference.**

"The Nursing Profession: How and Where to Train." 2s. net; post free 2s. 4d.
"Burdett's Official Nursing Directory." 3s. net; post free, 3s. 4d.
"Burdett's Hospitals and Charities." 5s.
"The Nurses' Dictionary of Medical Terms." 2s.
"Burdett's Series of Nursing Text-Books." 1s. each.
"A Handbook for Nurses." (Illustrated). 5s.
"Nursing: Its Theory and Practice." New Edition. 3s. 6d.
"Helps in Sickness and to Health." Fifteenth Thousand. 5s.
"The Physiological Feeding of Infants." 1s.
"The Physiological Nursery Chart." 1s.; post free, 1s. 3d.
"Hospital Expenditure: The Commissariat." 2s. 6d.

All these are published by the Scientific Press, Ltd., and may be obtained through any bookseller or direct from the publishers, 28 and 29 Southampton Street, London, W.C.

### Travel Notes.

By Our Travelling Correspondent.

### XCVII.—WHAT TO SEE ROUND AMSTERDAM.

There are several modest excursions to be made before attacking the larger ones of Hoorn Alkmaar, etc. . . . First to the Island of Marken, reached by steam tram to Monnickendam in less than an hour, and thence by steamer, or direct by steamer from Amsterdam itself. The island is very curious, and quite worth seeing, though guide-books take little account of its modest attractions. Baedeker devotes three lines to it, and those three lines would never lead one to visit it. It was a few words of Chantrey Churchill's that guided me to its old-world attractions:—"Those who love quaint costumes and customs will thoroughly enjoy a visit to the little Island of Marken, on the Zuyder Zee. Here, two miles from the main-land, the people still live the life of 200 years ago, and know nothing of those questions which are turning the rest of the world upside down. Socialism is unknown there, for every one is equal. Fashion has no slaves, for the dresses are handed down from generation to generation. . . . As the Marken housewife sits in her pretty home, which is as sweet and spotless as constant cleaning can make it, she forms a very pleasant picture. An elaborately embroidered bodice without sleeves is worn over a red and white striped shirt, the full sleeves of which cover the arms. On her head she wears a large white lace cap lined with brown (this lining serving the double purpose of showing off the pattern and keeping the lace clean); and beneath the cap her hair, parted in the middle and brushed smoothly to either side, hangs down behind in two plaits. . . . She never leaves the Island, and all the inhabitants are natives except the doctor and the clergyman. . . . The houses are made of wood and are gaily painted—green and black being the prevailing colours. The curious box bed forms the prominent feature in each interior, its snowy curtains and counterpane and elaborately decorated pillows making a very curious impression, and assuredly a much pleasanter one when viewed from the outside than from the in."

The men are all engaged in fishing, but the women do a good deal of very pretty drawn linen work. Sometimes the interiors show some fine old Delft ware, and a few good brass and copper utensils, but these are fast disappearing—sold to Jew traders and private collectors.

The Markenaars are kept primitive from their state of isolation, and it is still unusual for them to contract marriages with those from the mainland—foreigners, as they call them—but the increased facility of travelling will soon do away with this simplicity, and Marken will become as other places.

### The Hut of Peter the Great.

Six miles from Amsterdam is Zaandam, standing in a little forest of windmills. The chief attraction of the uninteresting little place is Peter's hut—admission 25 cents. It was here, in 1697, that the Czar lived whilst engaged in the shipbuilding yard of Kalf. It is now proved beyond doubt that he only occupied this place about a week, for he was recognised, and life became insupportable. The hut is now the property of the reigning Czar.

Broek, the cleanest village in the world. Truth compels me to say that this pharasaical little hamlet is extremely ugly. Painting and gilding reign everywhere; the Hollanders have a mania for paint, they can never have enough of it—the walls yellow or pink, the window shutters pea green, even the tree trunks painted as high as the lowest branches a starch blue or emerald green, the very earth painted for many yards round each homestead. The poor cows in their stalls pay the penalty of this mania for cleanliness, their tails being fixed up to rings lest the slightest soil should mar the Broek purity.

### Alkmaar.

Try to fix on a Friday if you visit the town only for a day, because being the weekly market there is so much more going on, and peasants in all kinds of quaint dresses come in from the neighbouring villages to buy and sell cheese. It takes three hours to reach Alkmaar by the canal, and it is much pleasanter than the rail. I suppose the first thing you will visit is the weigh-house, full of those magenta hued cheeses that we see in England. If you photograph you will be charmed with the variety of dress. The North Holland women still wear the strange gold helmet with two projections at the temples, sometimes spiral and something like a ram's horn, and sometimes more in the form of a rosette. Over this they wear a lace cap, and alas! of late years, they have taken to surmounting the whole with a bonnet and artificial flowers!

The weigh-house is the glory of Alkmaar, and the effect of its elaborate gabled roof and quaint front, reflected in the water below is delightful. All round in the Market square are queer-shaped home-made vehicles, small and squat, drawn by dogs, from one to three according to size, carrying milk, bread or vegetables, and everywhere mountains of cheeses, yellow and red. Inside the weigh-house, the municipal scales, which have been busy for centuries are still actively employed, whilst interested merchants with huge china-bowled pipes and anxious housewives in their golden headgear, watch the proceedings with interest.

It is strange to walk the streets of Alkmaar, now given up to buying and selling peaceful cheeses, to remember the deadly and heroic struggle that went on here in 1573, when the little city successfully resisted the besieging Spaniards for seven weeks. The inhabitants, with the awful fate of Haarlem fresh in their minds, determined to fight to the last, and had the courageous audacity to face 16,000 of Spain's best soldiers with a little force of 2,100 able-bodied men, of whom only 800 were soldiers. Twice the Spanish general attempted to carry the place by assault, and twice his troops were successfully repulsed. By the noble efforts of a carpenter in the city, Peter Van der Mey, who undertook the perilous task of passing through the enemy's lines, advice and assistance was asked of the Prince of Orange. His reply (enclosed in a walking stick) was that Alkmaar must hold out, but that when further resistance was impossible the citizens were to light certain beacon fires when the sluices and dykes should be opened (though this meant the ruin of the province) and the invading army must flee or be drowned! In returning Van der Mey was captured; he escaped with his life, but his stick was captured and examined and the dogged determination of the Dutch to be ruined rather than to submit and to involve themselves and their enemies in one common destruction determined the Spaniards to raise the siege, which was done after seven weeks' investment.

---

### TRAVEL NOTES AND QUERIES.

Cromer in May (Hygiene).—If you still are unsuited write to Miss Hicks, Baldock, Herts, who has a furnished cottage to let on reasonable terms—woman near who would act as servant. Two and a-half miles from Cromer and one and a-half from Sherringham.

Continental Holiday (Josephine).—Caudebec on the Seine is a charming spot, excursions all round and reasonable hotels. Return journey, second class, about £1 15s. Hotel de la Marine, 6 or 7 francs per day. You could just manage this for a month on £10 each, but it would be a close fit. If you only stay three weeks you would have plenty. See Nursing Mirror for March 25th, 1899, article "Up and Down the Seine." Belgium is rather cheaper than France. Dinant on the Meuse, second return, £1 19s. 11d. Living a trifle cheaper than Caudebec. Hotel des Familles 6 francs per day. Lovely country.

France for Good Accent (Nurse S.).—I do not think there is anywhere in France except a convent where two people could live for £30, much less £20, including their travelling expenses, for a month or six weeks. Paris or its outskirts not to be thought of. See answer to Josephine; that is the best I can think of. There is a convent where you could be received for about 22s. a week at Trequier in Brittany. Write to the Mother Superior and offer £1 a week. The only other place approaching this in cheapness is Knokke in Belgium. See Nursing Mirror for September 1st, 1900. As to accent, Belgium is not so good as France naturally, but with educated people it is always fairly good, and one cannot have everything. Unfortunately in travelling one seldom mixes much with the upper classes.

